# Toward a hierarchy of mechanisms in CaMKII-mediated arrhythmia

**DOI:** 10.3389/fphar.2014.00110

**Published:** 2014-05-16

**Authors:** Kevin P. Vincent, Andrew D. McCulloch, Andrew G. Edwards

**Affiliations:** ^1^Department of Bioengineering, University of California San DiegoLa Jolla, CA, USA; ^2^Department of Medicine, University of California San DiegoLa Jolla, CA, USA; ^3^Institute for Experimental Medicine, Oslo University Hospital UllevålOslo, Norway; ^4^Simula Research LaboratoryLysaker, Norway

**Keywords:** CaMKII, arrhythmias, afterdepolarizations, ryanodine receptor, cardiovascular diseases

## Abstract

Calcium/calmodulin-dependent protein kinase II (CaMKII) activity has been shown to contribute to arrhythmogenesis in a remarkably broad range of cardiac pathologies. Several of these involve significant structural and electrophysiologic remodeling, whereas others are due to specific channelopathies, and are not typically associated with arrhythmogenic changes to protein expression or cellular and tissue structure. The ability of CaMKII to contribute to arrhythmia across such a broad range of phenotypes suggests one of two interpretations regarding the role of CaMKII in cardiac arrhythmia: (1) some CaMKII-dependent mechanism is a common driver of arrhythmia irrespective of the specific etiology of the disease, or (2) these different etiologies expose different mechanisms by which CaMKII is capable of promoting arrhythmia. In this review, we dissect the available mechanistic evidence to explore these two possibilities and discuss how the various molecular actions of CaMKII promote arrhythmia in different pathophysiologic contexts.

## INTRODUCTION

**FIGURE 1:**
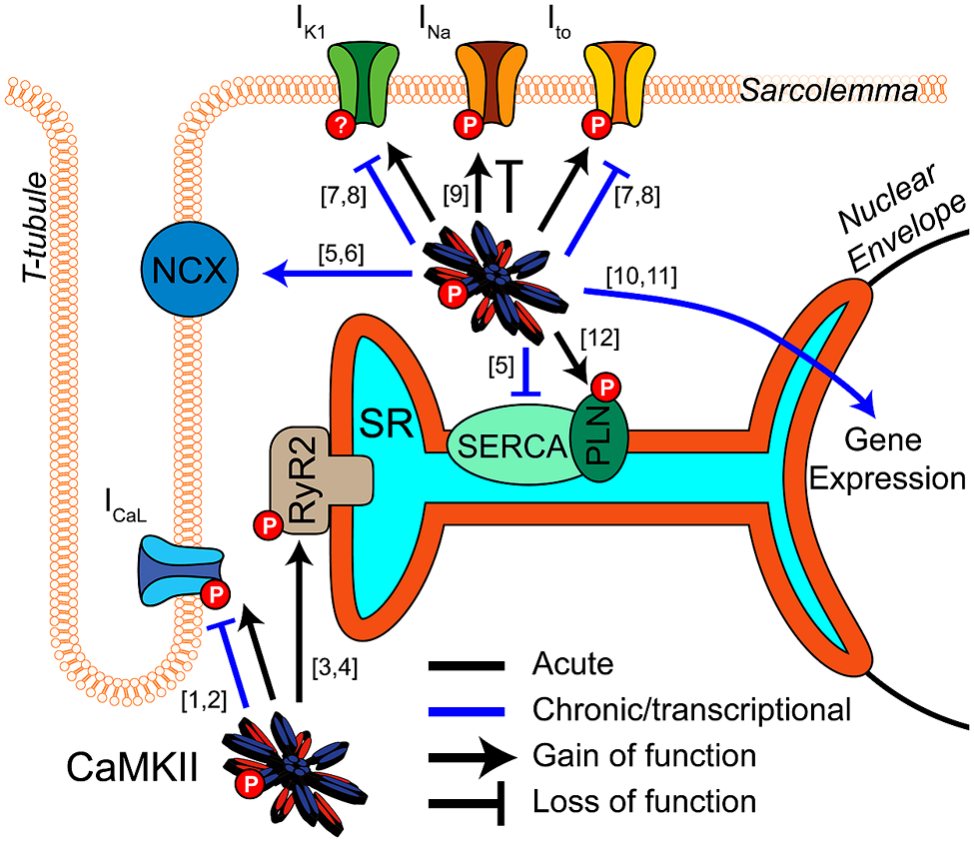
**CaMKII regulation of cardiomyocyte electrophysiology and Ca^2+^ handling.** CaMKII exerts acute (black) and transcriptional (blue) regulation ([10]-Wu et al., 2006; [11]-Backs et al., 2006) of many key proteins in cardiac electrophysiology and calcium handling. Acute phosphorylation of LCCs by CaMKII potentiates I_CaL_ and slows inactivation ([1]-Yuan and Bers, 1994). Transcriptional effects are less established but likely downregulate channel expression ([2]-Ronkainen et al., 2011). Phosphorylation of RyR2 by CaMKII promotes Ca^2+^ release from the SR and is implicated in many proarrhythmic contexts ([3]-Witcher et al., 1991; [4]-Wehrens et al., 2004). Available data suggest CaMKII transcriptional regulation promotes Ca^2+^ extrusion from the cell by increasing NCX expression and decreasing SERCA ([6]-Mani et al., 2010; [5]-Lu et al., 2011). I_to_ and I_K1_ are enhanced by acute CaMKII phosphorylation ([7]-Li et al., 2006), and transcriptional downregulation of these currents is a well-established effect of long term CaMKII activity. This reduces repolarization reserve and may destabilize resting membrane potential ([8]-Wagner et al., 2009). Acute regulatory effects of CaMKII on Na_V_1.5 enhance inactivation, decrease availability and potentiate I_NaL_, but again any transcriptional regulation is unclear ([9]-Wagner et al., 2006). Acute phosphorylation of PLN disinhibits SERCA and enhances SR Ca^2+^ reuptake ([12]-Karczewski et al., 1997).

Calcium/calmodulin-dependent protein kinase II (CaMKII) is a key regulator of excitation-contraction coupling in cardiac myocytes. As described in detail elsewhere in this special issue, CaMKII modulates the function and expression of numerous myocyte ion channels and calcium handling proteins (**Figure 1**). These include Na_V_1.5, the L-type Ca^2+^ channel (LCC), several potassium channel subunits, the cardiac isoform of the ryanodine receptor (RyR2), and phospholamban (PLN; Bers and Grandi, 2009). Many of the effects at these targets are proarrhythmic (Swaminathan et al., 2012; Fischer et al., 2013b), although some are antiarrhythmic by conventional paradigms (Tessier et al., 1999; El-Haou et al., 2009; Cheng et al., 2012), and therefore need to be considered in combination. Integrating these pleiotropic effects is further complicated by the temporal, spatial, and biochemical complexities of CaMKII activation (see articles herein from Erickson, 2014 and Gray and Heller Brown, 2014) in the intact myocyte. Thus, deciphering how CaMKII activity contributes to arrhythmia in any disease context is complex and non-intuitive.

These complexities notwithstanding, the potential for CaMKII inhibition to reduce arrhythmogenic outcomes has been demonstrated in animal models of a remarkably broad range of human diseases: from genetic channelopathies, such as catecholaminergic polymorphic tachycardia (CPVT), to etiologically complex pathologies such as heart failure, atrial fibrillation (AF), and ischemia/reperfusion injury (I/R). For this reason, and in addition to its potential as a target for anti-hypertrophic therapy, CaMKII has become a leading candidate for anti-arrhythmic targeting in the heart. Here we use this diverse range of disease models to develop a conceptual hierarchy of mechanisms in CaMKII-mediated arrhythmia. Specifically, we interrogate whether current evidence suggests that CaMKII promotes arrhythmia via multiple mechanisms, each of which can be recruited by particular disease etiologies, or alternatively, that CaMKII drives the same terminal mechanism irrespective of the specific underlying pathology. We contend that, while CaMKII disrupts ionic homeostasis and impairs repolarization through its actions at many different targets, the available evidence suggests that these mechanisms eventually converge to result in spontaneous Ca^2+^ release (SCR) from the sarcoplasmic reticulum (SR) and delayed afterdepolarizations (DADs). Thus we suggest that this is a dominant terminal mechanism of CaMKII-mediated arrhythmia, and may explain why experimental manipulation of CaMKII phosphorylation at RyR2 has proven effective in many models of arrhythmogenic disease.

## CELLULAR MECHANISMS OF CaMKII-MEDIATED ARRHYTHMIA

**FIGURE 2:**
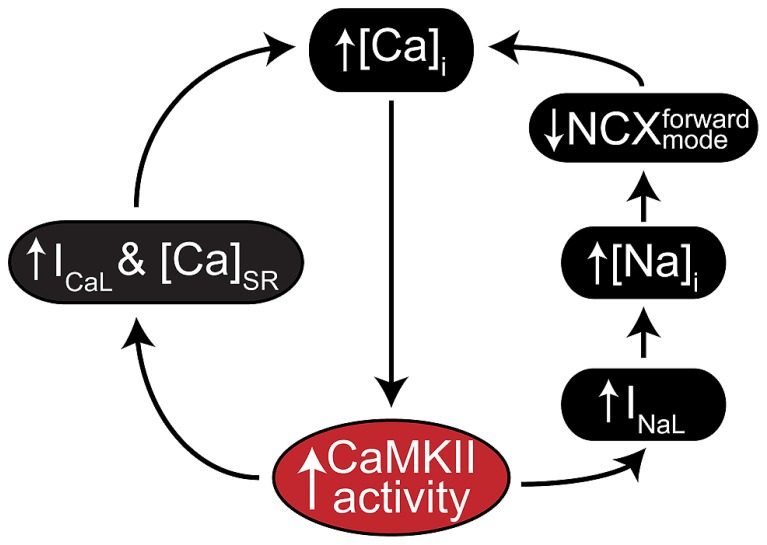
**CaMKII positive feedback mechanisms involved in cardiac disease.** As a Ca^2+^ regulated kinase, CaMKII is sensitive to any physiological mechanism that alters intracellular Ca^2+^ cycling. Many of its own catalytic actions result in such alterations, and two gain-of-function effects that create positive feedback by enhancing Ca^2+^ cycling occur at I_CaL_ (left) and I_Na_ (right). CaMKII regulation of I_Na_ is thought to increase intracellular Na^+^ via enhanced late I_Na_, which in turn reduces the thermodynamic potential for Ca^2+^ extrusion via NCX. CaMKII regulation of I_CaL_ more directly enhances Ca^2+^ cycling by increasing Ca^2+^ influx through slightly elevated peak current and slowed inactivation.

**FIGURE 3:**
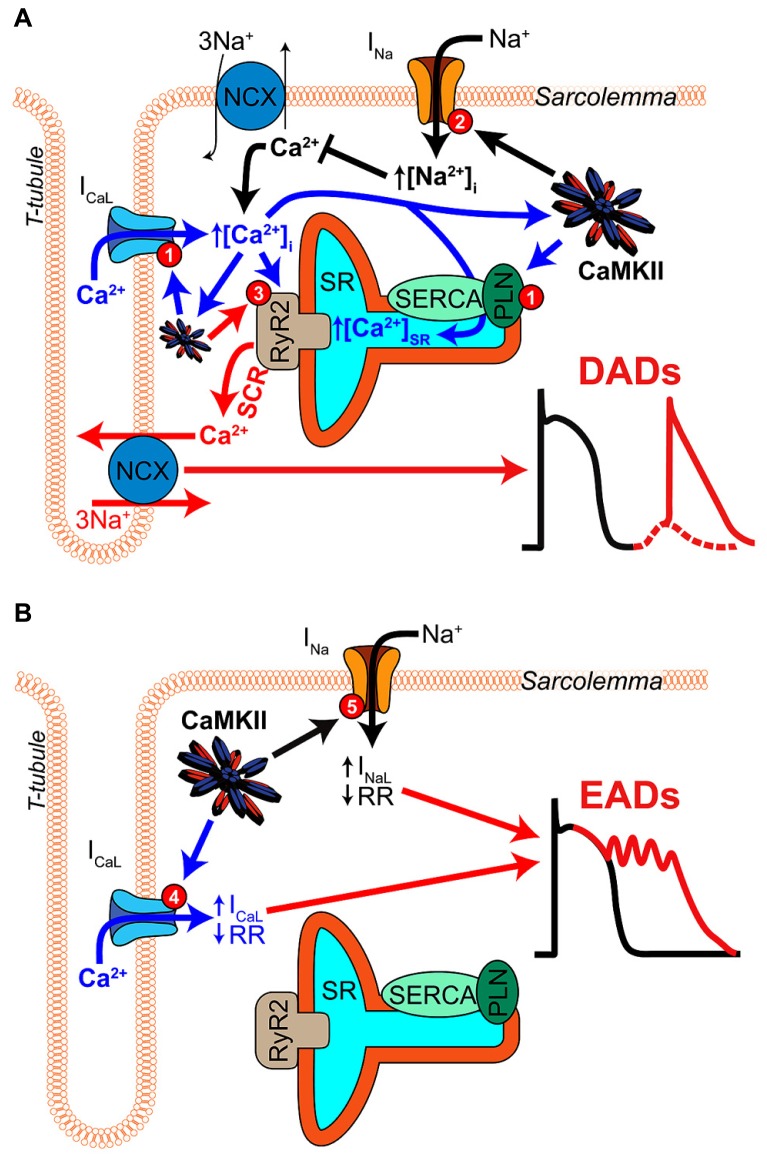
**Mechanisms of CaMKII-mediated afterdepolarizations. (A)** Pathological CaMKII regulation can trigger diastolic SR Ca^2+^ release resulting in electrogenic Ca^2+^ extrusion by NCX and DADs (red arrows). This results from pro-arrhythmic CaMKII regulation at multiple target proteins, which together drive 3 mechanisms through which CaMKII promotes SCR. First, CaMKII elicits gain-of-function effects at I_CaL_ and SERCA (via PLN-mediated disinhibition), thereby increasing Ca^2+^ influx and resulting in Ca^2+^ overload (mechanism 1, blue arrows). Second, CaMKII hyper-phosphorylation of Na_V_1.5 can elevate intracellular Na^+^ resulting in Na^+^-induced Ca^2+^-overload by decreasing Ca^2+^ efflux through NCX (mechanism 2, black arrows). Third, CaMKII directly phosphorylates RyR2, which has been shown to promote SCR and has been strongly implicated in a number of different models of arrhythmogenic disease (mechanism 3, red arrow). **(B)** CaMKII phosphorylation of depolarizing currents reduces repolarization reserve and promotes EADs (red arrows) in certain disease contexts. Altered LCC gating due to CaMKII hyper-activity can elicit EADs by increasing and prolonging I_CaL_ (mechanism 4, blue arrows). Na_V_1.5 phosphorylation increases I_NaL_ and may lead to non-equilibrium reactivation of I_Na_ (mechanism 5, black arrows), both of which can trigger EADs.

A key feature of CaMKII signaling in arrhythmia is the presence of several positive feedback mechanisms, whereby CaMKII target phosphorylation results in changes to Ca^2+^ homeostasis (or metabolism) that further activate the kinase. These mechanisms are likely to contribute both during acute homeostatic challenge, such as β-adrenergic stimulation, and to the chronic disruption of ionic homeostasis in acquired disease. **Figure 2** shows two major forms of this feedback. Hyper-phosphorylation of LCCs, PLN, (**Figure 3A**, mechanism 1) and Na_V_1.5 (**Figure 3A**, mechanism 2) increase whole-cell Ca^2+^ load either directly (LCC and PLN) or by impairing forward mode Na^+^/Ca^2+^ exchange (Na_V_1.5). This both promotes SCR in and of itself, and also further activates CaMKII, which independently drives SCR through phosphorylation of RyR2 (**Figure 3A**, mechanism 3; Wehrens et al., 2004). While it is not shown in **Figure 2**, the increased cytosolic Ca^2+^ also causes mitochondrial Ca^2+^ loading and probably invokes a third positive feedback loop involving ROS-dependent CaMKII activation (see Joiner and Koval, 2014 in this issue). Of course, the actions of CaMKII at LCCs, I_Na_, and certain potassium currents also directly result in proarrhythmic changes to the action potential (AP), including prolonged repolarization and early afterdepolarizations (EADs, **Figure 3B**; Guo et al., 2006). Thus, even at the level of integrated physiological outcomes, there are a number of CaMKII-dependent mechanisms capable of disrupting cardiac ionic homeostasis and electrophysiology. Our focus here is to understand which of these predominates in experimental models of human disease.

## ARRHYTHMOGENIC INFLUENCE OF CaMKII IN CONGENITAL HEART DISEASE

The last 20 years have witnessed rapid progress in understanding the molecular and genetic basis of congenital arrhythmogenic diseases (Curran et al., 1995; Cerrone et al., 2012; Napolitano et al., 2012). Somewhat surprisingly, CaMKII activity and hyperactivity have been shown to contribute in many experimental models of these diseases, even those with minimal or undetectable remodeling of myocardial structure. As such, these models offer a unique perspective of how CaMKII promotes arrhythmia in the absence of sequelae associated with structural disease. In this section, we review examples of CaMKII involvement in congenital arrhythmogenic disease, and identify key questions that remain for defining the roles, limits, and potential for therapeutic targeting of CaMKII in these contexts. Impressively, the available data suggest that CaMKII-dependent regulation of RyR2 is an important contributor to arrhythmogenesis in the majority of these congenital disease models. Here we focus specifically on how this regulation contributes to electrophysiologic instability, and suggest the review contributed by Camors and Valdivia (2014) for those interested in a more mechanistic description of CaMKII-dependent regulation of RyR2.

### CATECHOLAMINERGIC POLYMORPHIC VENTRICULAR TACHYCARDIA

The autosomal dominant form of CPVT has become the prototypical disease of RyR2-mediated arrhythmia, and accounts for at least half of all positive CPVT diagnoses (Priori et al., 2002). This channelopathy results from a family of RyR2 mutations that cause channel hyperactivity due to increased receptor Ca^2+^ sensitivity. The outstanding physiological ramification of these mutations is increased myocyte susceptibility to SCR and DADs during catecholamine-induced Ca^2+^ overload. As such, β-blocker therapy is a front-line treatment for CPVT patients. However, in ~50% of cases, episodes of VT still present during β-blockade, and more aggressive treatment (including ICD implantation) is often required (Priori et al., 2002; Watanabe and Knollmann, 2011). Thus, efforts to define additional effective pharmacotherapeutic approaches for this disease remain a high priority.

Conceptually, CaMKII presents an attractive target in CPVT because its established actions at various Ca^2+^ handling proteins would all be expected to exacerbate arrhythmia resulting from RyR2 hyperactivity. Indeed, transgenic overexpression of CaMKIIδ_C_ in the R4496C (+/-) mouse model of CPVT worsened the arrhythmia phenotype and increased mortality (Dybkova et al., 2011). However, few studies have assessed whether, and how, CaMKII activity contributes to arrhythmia in CPVT. Liu et al. (2011) showed that both chronic and acute administration of KN-93 dramatically reduced the incidence of arrhythmia in the R4496C (+/-) mouse. More specific CaMKII blockade by autocamtide-2 related inhibitory peptide (AIP) also markedly decreased the incidence of SCR, DADs, and triggered activity in isolated R4496C (+/-) ventricular myocytes (VMs). Interestingly, while AIP did remove signature effects of CaMKII on Ca^2+^ handling (e.g., frequency dependent acceleration of relaxation, FDAR), it did not achieve those effects by reducing SR Ca^2+^ load at any baseline pacing frequency or during isoproterenol (Iso) challenge. Thus, the antiarrhythmic actions of CaMKII blockade were unlikely to result from reduced SR or whole cell Ca^2+^ overload. Instead, they appeared to rely on reduction of CaMKII-induced RyR hyperactivity (**Figure 3A**, mechanism 3), as measured by triggered fractional release, and spark frequency. AIP reduced both of these measures in R4496C (+/-) myocytes, particularly during Iso challenge, and KN-93 reduced RyR2 phosphorylation at the established CaMKII site, Ser-2814. Thus, this study suggests that, even in myocytes already expressing hyperactive mutant RyR2, CaMKII phosphoregulation of this critical SR Ca^2+^ release channel may further contribute to *in vivo* arrhythmogenesis.

### CONGENITAL LONG QT SYNDROME

Recent work has suggested that CaMKII is involved in several rare forms of congenital long QT (LQT) syndrome. Importantly, CaMKII mutations have not been established as the source of genetic susceptibility in these, or to date, any congenital arrhythmogenic disease. Rather, in all existing studies, CaMKII exerts its proarrhythmic influence by exacerbating the effects of disease-associated mutations occurring in other electrophysiologic or calcium handling proteins, many of which are CaMKII targets.

#### Ankyrin-B syndrome/LQT4

After remaining elusive for over 10 years, the molecular basis for type 4 LQT syndrome was identified in 2003 as a family of loss-of-function mutations in Ankyrin-B (Mohler et al., 2003). The electrophysiologic dysfunction associated with these mutations is broad, and often also involves sinus bradycardia and catecholamine-induced arrhythmia, but is not associated with gross structural defects. LQT is relatively mild in most cases, and absent for some mutations even though individuals harboring these mutations remain arrhythmia susceptible (Mohler et al., 2004). Owing to this spectrum phenotype and clear molecular source, LQT4 is now often referred to as Ankyrin B syndrome (Yong et al., 2003). Heterozygous deletion of AnkB in the mouse recapitulates many signatures of human LQT4 (Mohler et al., 2003), including inducible polymorphic VT, and this model has now been used extensively to study mechanisms of the disease.

In neonatal AnkB(+/-) myocytes, LQT4 mutations consistently reduce expression and disrupt localization of the myocardial Na^+^/Ca^2+^ exchanger (NCX1), Na^+^/K^+^ ATPase (NKA), and inositol triphosphate (IP3) receptor (Mohler et al., 2003, 2004). In adult myocytes, these changes promote signature arrhythmogenic changes including AP prolongation (DeGrande et al., 2012), a prominent increase in Ca^2+^ waves (Camors et al., 2012) and afterdepolarizations (DeGrande et al., 2012). The mechanistic link between altered AnkB molecular anchoring and these arrhythmogenic outcomes is not fully established, but recent studies summarized below have suggested an important role for altered Ca^2+^ homeostasis, and particularly for CaMKII-dependent regulation of RyR2.

Given the observed changes to expression and distribution of major Na^+^ transporters, Camors et al. (2012) investigated whether altered Na^+^ handling could explain arrhythmogenicity in AnkB(+/-) myocytes. They found that, while maximal NKA function was depressed, this had little impact on measures of basal or challenged Na^+^ homeostasis, and did not increase diastolic [Ca^2+^]_i_. However, AnkB(+/-) myocytes did exhibit slightly increased SR Ca^2+^ load, enhanced Ca^2+^ transient amplitude and fractional release, and a marked increase in spark frequency and Ca^2+^ waves. Soon after, the same group was able to show that these effects are associated with RyR2 hyperactivity and increased phosphorylation of RyR2 at the primary CaMKII site, S2814 (DeGrande et al., 2012). These effects could be normalized by crossing the AnkB(+/-) mouse with one expressing the CaMKII inhibitory peptide, AC3-I. These mice were also resistant to both cellular afterdepolarizations and organ level arrhythmia present in the AnkB(+/-) mice during adrenergic challenge (DeGrande et al., 2012).

While the authors acknowledged that these RyR2 effects are one component of what are probably many alterations associated with AnkB loss-of function, the observed changes in Ca^2+^ handling are consistent with a mechanism involving RyR2 hyperactivity (Camors et al., 2012). Because AnkB is not known to anchor CaMKII itself, the authors instead suggested that loss of AnkB may have disrupted local phosphatase activity because protein phosphatase 2A is a known binding partner of AnkB and regulator of RyR2 phosphorylation.

#### Timothy syndrome/LQT8

The extremely rare LQT8, more frequently referred to as Timothy syndrome (TS), results from substitution mutations in the alpha subunit (Ca_V_1.2) of the cardiac L-type calcium current (I_CaL_). Only two mutations have been described to date, and both replace glycine residues with either arginine (pos. 406) or serine (pos. 402; Splawski et al., 2004, 2005). These mutations profoundly impair voltage-dependent inactivation of I_CaL_ (VDI), and this is generally accepted to be the proximal molecular dysfunction associated with the disease (Barrett and Tsien, 2008). The cardiac signatures of TS include a range of cardiac structural defects and markedly prolonged ventricular repolarization (Splawski et al., 2004, 2005). Supraventricular arrhythmia has also been noted, particularly AV block (Marks et al., 1995), but ventricular arrhythmogenesis is the most serious presentation of the disease, and most TS patients experience life-threatening events in the first years of life (Splawski et al., 2004). The existence and severity of the TS phenotype has forced clinicians and scientists to broadly reconsider the importance of VDI in normal and pathologic cardiac electrophysiology.

Since the original mechanistic descriptions from Splawski et al. (2004, 2005), a number of experimental models have been developed to investigate how these point mutations destabilize cardiac electrophysiology. In general, those models have provided compelling (albeit not entirely consistent) evidence for the involvement of CaMKII. Erxleben et al. (2006) were the first to suggest a role for CaMKII, and observed that heterologous expression of TS-mutated (G406R) rabbit Ca_V_1.2 yielded LCCs exhibiting more frequent prolonged openings (mode-2 gating). In noticing that this substitution also created a consensus site for CaMKII at nearby rabbit Ser-439 (homologous to human Ser-409), they hypothesized that CaMKII activity may be required for both mode-2 gating and slowed macroscopic inactivation of I_CaL_ in TS. CaMKII inhibition by KN-62, and genetic ablation of the putative CaMKII phosphoacceptor (S439A) indeed eliminated the increased intrinsic mode-2 openings of the mutant channels in that study. Thiel et al. (2008) extended this work to adult VMs by double-mutating Ca_V_1.2 to include both G406R and resistance to dihydropiridine inhibition via T1066Y. This permitted the authors to modulate the contribution of endogenous LCCs to macroscopic I_CaL_ via nifedipine, and thereby study the effects of the loss of I_CaL_ control, independent of differences in peak I_CaL_. Unlike Erxleben et al. (2006), they were unable to observe a requirement for CaMKII in the loss of VDI accompanying the G406R mutation. However, they did observe an interaction between VDI loss and CaMKII, whereby TS myocytes exhibited greater I_Ca_ facilitation than WT, and this could be removed through peptide blockade of CaMKII via AC3-I. To explain this they suggested that Ca^2+^ loading due to impaired VDI caused enhanced secondary activation of CaMKII, which in turn exaggerated I_Ca_ facilitation. They also observed a pronounced increase in SCR and DADs, both of which could be prevented by AC3-I. As a result they reasoned that DADs occurring secondary to Ca^2+^ overload may reflect the dominant arrhythmogenic role of CaMKII in TS, and mathematical modeling supported this contention. Thus, in VMs, CaMKII activation may compound the effects of TS mutations by enhancing I_CaL_ facilitation in response to impaired VDI and increased Ca^2+^ influx.

Further cardiac-specific models of TS have now been developed, most notably the G436R transgenic mouse developed by Cheng et al. (2011). This model exhibits increased Ca_V_1.2 expression, and the entirety of this increase (~40%) is due to expression of the G436R transgene. Somewhat surprisingly this additional Ca_V_1.2 does not increase peak I_CaL_ in these mice but does slow VDI in the expected manner, and intriguingly, this effect required the anchoring protein AKAP150. To explain this result, the authors suggest that AKAP150 provides a key structural link among LCCs within the dyadic ensemble, and they provide evidence that, in the presence of G436R-mutated channels, AKAP150 promotes coupled gating of LCCs and increases mode-2 behavior. Similarly to Thiel et al. (2008), Yarotskyy et al. (2009), and Cheng et al. (2011) observed that slowed inactivation in this model is not impacted by CaMKII inhibition, and therefore suggest that the gating effects underlying TS are probably not reliant upon G436R-mediated creation of a CaMKII phosphorylation site at Ser-439/409. They did, however, observe markedly enhanced Ca^2+^ cycling, prolonged AP duration, and exaggerated susceptibility to cellular afterdepolarizations. These effects were confirmed in a recent and more detailed study of Ca^2+^ handling in these mice (Drum et al., 2014), and again like Thiel et al. (2008) the authors conclude that Ca^2+^ overload and SCR are the likely terminal mechanisms of arrhythmogenesis in this model.

In summary, these investigations suggest that CaMKII plays an important role in TS arrhythmogenesis, but at least in VMs it seems probable that this accompanies Ca^2+^ overload secondary to the intrinsic effects of the TS mutations on LCC gating. An important qualifier here is that, to date, all experiments suggesting this role for CaMKII have been conducted in primary cardiomyocytes from rodents. The rapid repolarization of these cells probably biases arrhythmogenic mechanisms away from disrupted repolarization and toward SCR and arrhythmia arising during diastole. Thus, similarly detailed investigations in large mammalian myocytes remain desirable for clarifying if changes in I_CaL_ gating are capable of playing a more direct role in human TS arrhythmia, and if CaMKII still contributes in a similar manner and to a similar degree.

#### Phosphomimetic mutation of Na_V_1.5

CaMKII is an established regulator of the myocardial Na^+^ current (I_Na_), and simultaneously potentiates late I_Na_ (I_NaL_) while decreasing channel availability (Herren et al., 2013). While the specific phosphorylations required for these effects remain debated, available evidence suggests that either or both Ser-571 and Ser-516 may be key sites (Hund et al., 2010; Ashpole et al., 2012; Koval et al., 2012; Herren et al., 2013), and it is generally agreed that the I-II intracellular linker is the critical phosphoregulatory domain.

An interesting line of investigation has suggested that mutation of residues immediately adjacent to Ser-571 are associated with congenital disease, and may result in phosphomimetic effects at I_Na_. Two mutations, A572D and Q573E, were originally uncovered by genetic screening of Romano-Ward syndrome probands, and therefore thought to be novel forms of LQT3 (Paulussen et al., 2003; Tester et al., 2005). The A572D mutation has since been dismissed as an independent source of LQT3 susceptibility, but may still be associated with arrhythmogenic cardiac diseases because it cosegregates with the established arrhythmogenic mutation H558R (Tester et al., 2010). Having previously identified Ser-571 as a potential site for CaMKII regulation, Koval et al. (2012) recently investigated whether the functional effects of these mutations exhibited any dependence on CaMKII activity. They showed that heterologous expression of either A572D or Q573E recapitulated CaMKII-dependent effects at I_Na_. Neither constitutively active CaMKII nor blockade of endogenous CaMKII altered these effects, suggesting that they are autonomous to the mutant Na_V_1.5 and independent of additional CaMKII phosphoregulation. Further, the mutant gating defects could be reconstituted in primary mouse VMs transfected with channels sensitive to low dose tetrodotoxin to allow silencing of the exogenous Na_V_1.5. While conflicting results from other groups (Tester et al., 2010; Ashpole et al., 2012) suggest that these findings require further interrogation, they provide the first evidence that CaMKII regulation of I_Na_ may be sufficient to induce an arrhythmogenic phenotype in the absence of structural or ischemic disease.

## ARRHYTHMOGENIC INFLUENCE OF CaMKII IN ACQUIRED HEART DISEASE

An extensive literature describes the arrhythmogenic mechanisms attributable to CaMKII hyperactivity in acquired heart disease. A large portion of this is related to heart failure, but significant effort has also been invested in AF and ischemia/reperfusion challenge (I/R). The CaMKII-dependent mechanisms involved in AF and I/R are detailed separately in this issue by Heijman et al. (2014) and Bell et al. (2014) and we direct the interested reader to those comprehensive treatments. For the purposes of this review it suffices to note that recent studies of AF and I/R mechanisms in mice and humans have again implicated RyR2 dysregulation, and SR Ca^2+^ handling in general (Said et al., 2008, 2011; Voigt et al., 2012; Purohit et al., 2013). These aspects are consistent among the various acquired disease even though other important pathological conditions differ. For example, some signature electrophysiologic alterations apparent in AF (Nattel et al., 2008), such as shortened APD and more negative resting potential, are precisely the opposite of the alterations present in failing VMs (Tomaselli and Marbán, 1999).

As might be expected of the pathologic complexity in acquired disease, the range of CaMKII-dependent mechanisms thought to contribute to arrhythmia is also broader than for congenital disease. Specifically, CaMKII has been shown to contribute to Na^+^ overload in heart failure (**Figure 3A**, mechanism 2), and several studies have suggested that CaMKII effects at I_CaL_ may be sufficient to directly destabilize electrophysiology in the failing ventricle (**Figures 3A,B**). An additional feature of CaMKII-dependent arrhythmia in heart failure and AF, and probably in acquired disease in general, is the importance of CaMKII-oxidation as a source of kinase hyperactivity (Luczak and Anderson, 2014; see also the review by Erickson, 2014 in this special issue). This mode of CaMKII activation appears to be particularly important in sinus node (SN) dysfunction accompanying heart failure (Swaminathan et al., 2011), in AF (Purohit et al., 2013), and diabetic cardiomyopathy (Luo et al., 2013). Finally, one of the most important roles that CaMKII plays in acquired disease is as a controller of the expression of several key ion channels and transporters, and many disease-associated changes in expression of these proteins appear to require CaMKII. The best-described of these involve K^+^ currents, namely the transient outward current (I_to_) and inward rectifier current (I_K1_; Wagner et al., 2009; reviewed elsewhere in this issue by Mustroph et al., 2014), but this transcriptional regulation may also extend to I_CaL_ (Ronkainen et al., 2011), NCX1 (Mani et al., 2010), and SERCA2 (Lu et al., 2011).

### HEART FAILURE

The role of CaMKII in heart failure pathophysiology has been the subject of intense investigation since elevated CaMKII levels were first found in the myocardial tissue of heart failure patients (Hoch et al., 1999; Kirchhefer et al., 1999). Increased CaMKII expression and activity have since been mechanistically linked to structural and electrophysiological dysfunction in numerous experimental models of severe cardiomyopathy and heart failure. However, deciphering precisely how CaMKII is driving electrophysiologic dysfunction is made all the more difficult by the extensive structural and electrophysiologic remodeling that accompanies CaMKII hyperactivity in the failing heart.

#### Target-specific roles of CaMKII in heart failure

Murine models of heart failure, CaMKII hyperactivity (overexpression), and CaMKII regulation of RyR2 have provided a wealth of information describing how CaMKII contributes to both arrhythmogenesis and disease progression in cardiomyopathy. Cardiac restricted overexpression of CaMKIIδ_C_ in the mouse leads to heart failure, inducible arrhythmia, and premature death (Zhang et al., 2003; Wagner et al., 2006). In these mice, acute inhibition of CaMKII (via KN-93) prevents catecholaminergic arrhythmia *in vivo*, and RyR2 dysfunction was implicated in this arrhymogenic mechanism (**Figure 3**, mechanism 3) by a substantial and CaMKII-dependent increase in SR Ca^2+^ leak, elevated diastolic Ca^2+^, and DADs during Iso challenge (Sag et al., 2009). SR-targeted inhibition of CaMKII with SR-AIP restored calcium handling but worsened the heart failure phenotype suggesting that other CaMKII mechanisms are integral to pathological remodeling (Huke et al., 2011). The phosphomimetic (S2814D) and non-phosphorylatable (S2814A) RyR2 mutants developed by Wehrens et al. (2004), have provided a powerful model for investigating the role of CaMKII-dependent RyR2 regulation in a large number of acute and chronic diseases. Most recently, the knock-in mice expressing these engineered RyR2 variants have provided compelling evidence that CaMKII regulation of this protein contributes to arrhythmogenesis in several models of acquired disease. The S2814D mouse slowly develops a heart failure phenotype and is susceptible to sustained VT during epinephrine/caffeine challenge, and pressure-overload initiated prior to overt heart failure also increased mortality due to arrhythmias in these mice (van Oort et al., 2010). Genetic ablation of CaMKII-dependent RyR2 phosphorylation in the S2814A mouse provides protection from pacing-induced arrhythmias after pressure-overload, slows the development of contractile dysfunction, and reduces ventricular remodeling and cellular SCR (van Oort et al., 2010; Respress et al., 2012).

CaMKII has also been shown to contribute to heart failure arrhythmias in large mammals. A series of studies in a rabbit model of non-ischemic heart failure provides further mechanistic evidence of the importance of CaMKII regulation of RyR2 in heart failure arrhythmogenesis. In this model, total CaMKII expression was elevated and the amount and activity of CaMKII localized to RyR2 was increased in failing ventricular tissue, thus leading to enhanced CaMKII-dependent phosphorylation of RyR2 (Ai et al., 2005). Elevated SR Ca^2+^ leak was significantly reduced with inhibition of CaMKII but not PKA (Ai et al., 2005), and Iso-induced SCR events (Ca^2+^ waves) were CaMKII-dependent (Curran et al., 2010). Belevych et al. (2011) also described progressive pathological RyR2 dysfunction in a tachycardia-induced canine model of heart failure. Mirroring changes in SR Ca^2+^ leak observed in the rabbit, phosphorylation of RyR2 by CaMKII but not PKA was increased after 1- and 16-months of tachycardic pacing. CaMKII inhibition with KN-93 abolished proarrhythmic diastolic calcium waves after 1 month but not 16-months. Instead, the increased wave frequency at 16 months was attributed to increased ROS production, which highlights the progressive nature of arrhythmia mechanisms in heart failure, and suggests that CaMKII hyperactivity may contribute more greatly to arrhythmogenesis early in heart failure progression. Significantly, the clinical importance of CaMKII modulation of RyR2 was reinforced recently when Fischer et al. (2013a) demonstrated that CaMKII inhibition with KN-93 or AIP reduced SR Ca^2+^ leak in myocytes isolated from failing human hearts.

CaMKII regulation of cardiac Na^+^ currents is another potential source of arrhythmogenic regulation during heart failure. The mechanisms of this regulation are presented briefly in **Figures 2** and **3** and in detail elsewhere in this issue (see Grandi and Herren, 2014), thus we only briefly mention them in the context of their importance in heart failure. I_NaL_ is increased in human heart failure (Valdivia et al., 2005), and evidence from numerous animal models suggests CaMKII inhibition normalizes I_NaL_. In a murine TAC-induced heart failure model, CaMKII inhibition by AIP reversed increases in I_NaL_, APD_90_, Iso-induced DAD frequency, and SR Ca^2+^ leak (Toischer et al., 2013). In that study, CaMKII phosphorylation of Na_V_1.5 was increased during decompensated heart failure and application of ranolazine had a similar result to CaMKII inhibition suggesting an important role for CaMKII regulation of Na^+^ handling. Interestingly, myocytes from pressure-overloaded hearts did not exhibit EADs in that study, suggesting that Na^+^ does not carry the arrhythmogenic current. Instead, the authors suggested that I_NaL_ was indirectly responsible for DADs by causing Na^+^-induced Ca^2+^-overload (**Figure 2**). This idea is consistent with reports from several other studies. Most recently Morotti et al. (2014) have suggested that Na^+^-induced Ca^2+^-overload is a component of a positive feedback loop that is quantitatively capable of eliciting CaMKII hyperactivity, which in turn promotes arrhythmogenic outcomes and, probably, further Na^+^ loading (**Figure 2**). Indeed, experiments involving Na^+^ overload induced by anemone toxin (ATX-II, a potent agonist of I_NaL_) and an LQT3 mutation (N1325S), have shown these maneuvers to be capable of inducing CaMKII activation and arrhythmia (Yao et al., 2011). Similar effects have been observed during glycoside-induced Na^+^ overload (Gonano et al., 2011; Ho et al., 2013), and arrhythmias resulting from glycoside treatment were susceptible to both SR-targeted peptide inhibition of CaMKII (Gonano et al., 2011) and ablation of CaMKII phosphorylation at RyR2 via S2814A mutagenesis (Ho et al., 2013). Thus, it may again be that these Na^+^-dependent forms of arrhythmogenesis converge at CaMKII-dependent regulation of RyR2. It should be noted that some evidence in large mammals also supports a more direct arrhythmogenic role for CaMKII-dependent regulation of Na^+^ currents (**Figure 3B**). In a canine model of dyssynchronous heart failure with extensive electrophysiological remodeling (Aiba et al., 2009), CaMKII expression, activity and autophosphorylation were all increased, particularly in the late-activated lateral LV wall (Chakir et al., 2008). Myocytes isolated from these dogs exhibited shifted voltage-dependent availability, enhanced intermediate inactivation, increased I_NaL_ and ranolazine sensitive EADs (Aiba et al., 2013). Although inhibition studies were not performed in these dogs, Maltsev et al. (2008) had previously identified CaMKII as an important regulator of I_NaL_ in the failing canine heart.

Regulation of LCCs by CaMKII presents a third potential mechanism for proarrhythmic function during heart failure. However, as for I_Na_ the sites of CaMKII phosphorylation of LCCs remain somewhat controversial, and this has caused mutagenic approaches to investigating LCC-dependent mechanisms to be more challenging. Similarly to TS, CaMKII regulation of I_CaL_ in heart failure is thought to elicit arrhythmia either by disrupting repolarization and driving EADs (**Figure 3B**), or by promoting cellular Ca^2+^ overload (**Figures 2** and **3A**). Again, a complete treatment of CaMKII effects at I_Ca_ is provided elsewhere in this issue (see the review from Bers), but briefly, heart failure-associated changes to LCC gating are quite similar to those accompanying CaMKII regulation (Schröder et al., 1998; Dzhura et al., 2000). Specifically, CaMKII elicits mode-2 gating in single channels, and slows inactivation while hastening recovery from inactivation of the macroscopic current (Guo et al., 2006). All of these effects would be expected to promote EADs by reducing repolarization reserve, independently promoting I_CaL_ reactivation, or both (Anderson et al., 1994; Hashambhoy et al., 2010; Koval et al., 2010). The most direct evidence for the ability of CaMKII to elicit EADs via I_CaL_ (in large mammal VMs), was presented by Koval et al. (2010). In that study, the Ca_V_1.2 β_2a_ regulatory subunit was expressed in rabbit VMs, which increased I_Ca_ facilitation and promoted EADs. Those effects were reversed by peptide (CaMKIIN) or shRNA inhibition of CaMKII, and ablation of the proposed CaMKII phosphorylation or binding sites (Koval et al., 2010). Two caveats when extending these findings to CaMKII hyperactivity in normal or failing myocytes are: (1) the β_2a_ subunit is probably a lesser component of LCCs in non-diseased hearts, although its expression may be increased in heart failure (Hullin et al., 2007), and (2) the slowed inactivation in this study also increased total Ca^2+^ influx without destabilizing the AP. *In vivo*, the effect of this on whole-cell and SR Ca^2+^ overload could easily be as or more important than effects upon repolarization.

Together these studies suggest that CaMKII-mediated ventricular DADs and EADs exert a proarrhythmic influence in the failing heart, and that the specific mode of arrhythmia initiation and maintenance is likely dictated by the underlying etiology and stage of disease development. The genetic tools available for studying the role of CaMKII regulation of RyR2 in mice have provided compelling evidence that this target is central to the acute arrhythmogenic outcomes of CaMKII in the failing ventricle, and this contention is largely supported by studies in large mammals. Given the extensive structural and electrophysiological remodeling accompanying heart failure, and relatively poor specificity of small molecule inhibitors available for *in vivo* CaMKII blockade, it is still not entirely clear how effective acute CaMKII inhibition may be as an anti-arrhythmic strategy in heart failure. However, evidence published to date support an optimistic outlook for potential therapeutic applications in this and other acquired cardiac diseases.

#### Sinus node dysfunction in heart failure

Another series of recent studies, reviewed in this issue by Wu and Anderson (2014), has suggested that oxidized-CaMKII (ox-CaMKII) plays a critical role in SN dysfunction accompanying heart failure, and that this involves pronounced structural remodeling of the SN. Swaminathan et al. (2011) first observed that, relative to non-diseased controls and heart failure patients without SN dysfunction, heart failure patients with SN dysfunction exhibit increased ox-CaMKII in right atrial tissue. This comprehensive study observed an analogous effect in a canine model of pacing-induced heart failure, and utilized their previously developed model of Ang II-dependent oxidation/activation of CaMKII to elicit and study the mechanisms of SN dysfunction in mice. With this model ~70% of mice develop sinus pause or exit block after 3 weeks of Ang II infusion, and heart rate at rest and during activity is blunted compared to untreated controls. SN dysfunction was absent in AC3-I (cardiac specific) mice treated with Ang II even though the pressor response to Ang II was similar to WT. The observed dysfunction was associated with increased apoptosis and fibrosis within the SN, and structurally based computational analysis suggests that the cell loss observed experimentally may be sufficient to explain both reduced SN firing frequency, and episodes of exit block. One particularly elegant component of this study showed that local adenoviral gene transfer of the CaMKIIN inhibitory peptide (via painting of the SN) was capable of preventing Ang II-driven SN dysfunction in WT mice. Thus providing proof-of-principle that gene transfer of CaMKII-inhibitors may be a viable therapeutic avenue for this specific heart failure phenotype. A follow-up study from Luo et al. (2013) investigating SN dysfunction in a mouse model of combined insulin deficiency and myocardial infarction, suggests that the mechanistic cascade described by Swaminathan et al. (2011), may extend to other cardiac conditions exhibiting pronounced oxidative stress.

The role of oxidized CaMKII in driving structural remodeling and dysfunction of the SN in response to oxidative stress highlights the ability of CaMKII to impact arrhythmogenic outcomes via structural as well as functional changes. To date, the impact of these tissue-level effects in CaMKII-mediated arrhythmia has not been studied to the same extent as the effects on cellular electrophysiology and Ca^2+^ handling. This aspect of CaMKII-driven arrhythmia warrants further work, as some evidence suggests that effects at this level may be important. For example, CaMKII activity may exhibit regional heterogeneity in the heart (Chakir et al., 2008). Even without considering this heterogeneity of kinase action, transmural dispersion of repolarization is a predicted result of CaMKII hyperactivity (Bers and Grandi, 2009) due exclusively to its effects at I_to_. Thus, putative roles for CaMKII in reentrant or alternans-driven arrhythmias remain largely untested.

## SUMMARY AND CONCLUSION

The ability of CaMKII to contribute to arrhythmia in models of cardiac disease that result from widely varying etiologies is testament to the importance of this kinase in the control of cardiac electrophysiology and calcium handling. It also suggests that CaMKII exerts its proarrhythmic influence either by regulating some convergent mechanism that is active in all of these diseases, or by regulating a divergent range of proarrhythmic mechanisms, which contribute to differing degrees in each disease. To date, the available mechanistic evidence suggests that CaMKII-dependent regulation of diastolic SR Ca^2+^ release is the dominant cellular mechanism by which the kinase promotes arrhythmia (**Figure 3A**). Of the various CaMKII targets capable of driving this mechanism, RyR2 is the most clearly implicated. This may in part be due to the quality of the tools available for genetically manipulating CaMKII phosphorylation and regulation of RyR2, but it also suggests that developing exogenous compounds capable of impairing the ability of CaMKII to phosphorylate RyR2 could constitute a broadly applicable therapeutic strategy. Additional effects of CaMKII upon I_Na_ and I_CaL_ may also directly contribute to arrhythmia in some contexts (**Figure 3B**), but in several of the examples described above it appears likely that these effects again converge to elicit arrhythmia via SCR and DADs (**Figure 3A**).

Both SCR and DADs are generally induced by some form of acute Ca^2+^ overload, and it is probable that this partially explains why CaMKII-dependent arrhythmia most often requires conditions of tachycardia and, notably, β-adrenergic challenge. The involvement of this additional signaling cascade brings in further complexity that is almost certainly important in defining the transition from stable to unstable electrophysiology. As suggested by studies involving the Ankyrin B (+/-) mouse (DeGrande et al., 2012), this may depend on less well-understood aspects of the signaling network, such as local phosphatase balance. As such, strong conclusions regarding any one CaMKII target as being crucial for broad arrhythmia phenotypes should remain contentious until these finer details have been described with some clarity.

Tissue-level effects of CaMKII are also undeniably important, as highlighted by SN dysfunction in heart failure, but beyond these investigations, little direct evidence is available to describe how CaMKII disrupts normal propagation of the cardiac AP. By incorporating regional heterogeneities in cellular electrophysiology, computational modeling may aide in understanding these tissue level effects provided sufficient experimental data for parameterization and validation are available. The reviews by Onal et al. (2014), Greenstein et al. (2014), and Yaniv and Maltsev (2014) offer a comprehensive survey of previous work modeling CaMKII on the cellular and tissue levels.

## CAVEATS AND IMPORTANT FUTURE DIRECTIONS

Even with the wealth of information that has amassed to describe the role of CaMKII in cardiac arrhythmia over the last 20 years, several key aspects either remain challenging or are otherwise conspicuously absent from the existing literature. First, no study has been capable of identifying a gain-of-function mutation in CaMKII that is an independent risk for arrhythmogenic cardiac disease, or indeed, any form of cardiac disease. This is surprising given the data available from studies involving the various CaMKII inhibitors. Second, a key unrealized objective of the CaMKII field is to have more specific CaMKII inhibitors that can be applied acutely and *in vivo* (see the review from Pellicena and Schulman, 2014 in this special issue). This is particularly true for studies of arrhythmia, where such inhibitors would allow more straightforward translation between cell-based experiments and arrhythmia outcomes in intact animals, and perhaps eventually humans. Indeed, as mentioned above, the tissue-level effects of CaMKII activity and hyperactivity are poorly understood compared to the cellular effects, and one reason for this is the lack of suitably specific small molecule inhibitors. Third, as mentioned with respect to TS, the use of murine myocytes for cell-based arrhythmia assays probably biases the active mechanisms away from those occurring during repolarization (EADs), and toward those that are active during diastole (DADs). This is because the rapidly repolarizing murine myocyte has markedly increased repolarization reserve, and current dynamics during repolarization that are at least very different to those in large mammals and humans (Nerbonne, 2004). As techniques for differentiating and reprogramming h-IPSCs improve, models based on this approach may offer a useful new tool for studying the role of CaMKII in congenital arrhythmogenic diseases at least. Examples of early studies in this direction have recently appeared for CPVT (Di Pasquale et al., 2013) and TS (Yazawa et al., 2011). However, concerns surrounding how well these cells recapitulate adult cardiomyocyte Ca^2+^ handling are particularly poignant in these and other Ca^2+^-related arrhythmogenic diseases. As such, the evidence supporting myocyte-specific differentiation should be considered carefully and specifically for any such future model.

## Conflict of Interest Statement

The authors declare that the research was conducted in the absence of any commercial or financial relationships that could be construed as a potential conflict of interest.
